# Voriconazole-Induced Periostitis & Enthesopathy in Solid Organ Transplant Patients: Case Reports

**DOI:** 10.4236/jbm.2016.411002

**Published:** 2016-11-10

**Authors:** Monica Sircar, Camille Kotton, David Wojciechowski, Kassem Safa, Hannah Gilligan, Eliot Heher, Winfred Williams, Ravi Thadhani, Nina Tolkoff-Rubin

**Affiliations:** 1 Division of Nephrology, Department of Medicine, Massachusetts General Hospital, Boston, MA, USA; 2 Division of Infectious Disease, Department of Medicine, Massachusetts General Hospital, Boston, MA, USA; 3 MGH Transplant Center, Departments of Medicine and Surgery, Massachusetts General Hospital, Boston, MA, USA

**Keywords:** Voriconazole, Periostitis, Organ Transplant, Enthesopathy, Drug Metabolism

## Abstract

**Background:**

Voriconazole is frequently used to treat fungal infections in solid organ transplant patients. Recently, there have been reports suggesting that prolonged voriconazole therapy may lead to periostitis.

**Aim:**

Here we present two cases of voriconazole-induced periostitis in solid organ transplant patients.

**Case Presentation:**

Voriconazole was given to two transplant patients-one with a liver transplant and the second with a heart transplant, to treat their fungal infections. Both developed voriconazole-induced toxicity. While undergoing voriconazole therapy, they had incapacitating bone pain. The liver transplant patient had to be taken off voriconazole, and the heart transplant patient succumbed to non-voriconazole related causes.

**Conclusions:**

Voriconazole therapy in two solid organ transplant patients resulted in periostitis. We provide potential etiologies underlying voriconazole-induced periostitis, including fluoride toxicity, abnormalities in the pulmonary vascular bed leading to the production of downstream inflammatory mediators, and abnormal pharmacokinetics of hepatic drug metabolism. In addition to monitoring blood voriconazole trough levels, we suggest careful assessment for musculoskeletal pain in patients undergoing voriconazole treatment for two months or more, particularly if their daily dosages of voriconazole exceed 500 mg per day. Appropriate workup should include measurement of alkaline phosphatase, voriconazole trough and fluoride levels as well as a bone scan. Overall, early recognition of voriconazole-induced musculoskeletal toxicity is important for better morbidity outcomes.

## 1.Introduction

Voriconazole is the drug of choice in the treatment of aspergillosis and other fungal infections seen in immunosuppressed transplant patients. In 2009, Wang and colleagues first described five cases of periostitis in lung transplant recipients secondary to prolonged voriconazole therapy [1].

Voriconazole-induced musculoskeletal toxicity has three distinguishing features. First, the patient typically presents with diffuse bone pain, frequently involving the ribs, shoulders and hips. Second, the patient has an elevated alkaline phosphatase level, at least three-fold higher than the upper limit seen in normal subjects. Finally, radionuclide imaging usually reveals bone inflammation [2]. Abnormalities have been described as “dense”, “symmetric”, and “feathery” in appearance, and are usually seen in the distal diaphysis of long bones, ulna and ribs [3]. In some cases, abnormalities can extend to the metaphysis and beyond [1]. The duration of voriconazole exposure prior to disease onset usually ranges between six and twenty-six months [1] [2] [4]-[22]. One case report described onset at six weeks [22].

## 2.Case Reports

The two case studies presented in this report highlight the unique aspects of musculoskeletal toxicity following voriconazole use. Details of each case are provided in Table 1.

### 2.1.Case 1

Patient was a 56 year-old Caucasian male with a history of alcohol dependence, hepatitis C and cryoglobulinemia. In April of 2013, he underwent an orthotopic liver transplant. His post-transplant course was complicated by persistent low-grade fevers. He underwent cerebral imaging (CT and MRI). Brain imaging revealed bilateral rim-enhancing lesions in the temporal lobes. There was evidence of edema suggesting the presence of abscesses. The patient underwent a stereotactic brain biopsy. Pathologic examination demonstrated *Scedosporium apiospermum* infection. The serum galactomannan level was negative. The patient was started on AmBisome^®^ (minimal inhibitory concentration (or MIC) of 4) and weight-based voriconazole (400 mg PO BID). Since further microbial investigation showed that the microbe was sensitive only to voriconazole, AmBisome^®^ was discontinued. Based on the infectious burden and immuno-compromised state of the patient, the treatment plan was to continue him indefinitely on voriconazole.

Approximately two months after transplant, the patient was re-admitted to the hospital with generalized fatigue and diffuse bone pain, specifically in the joints and ribs. He did not have any fever or chills. Blood and urine cultures were unrevealing. Non-contrast head CT revealed improvement in his cerebral fluid collection. Rheumatoid factor, anticyclic citrullinated peptide antibody (anti-CCP Ab), a biomarker highly-specific for rheumatoid arthritis, and double-stranded anti-nuclear antibody (anti-dsDNA) levels in blood were negative. Antinuclear antibody (ANA) was positive at 1:40. Complements C3 and C4 as well as parathyroid levels were normal. Parvovirus serology was consistent with prior infection. The patient's fluoride level was >24 umol/L (normal range ≤ 4 umol/L). Bone imaging showed evidence of periostitis.

**Figure 1:**
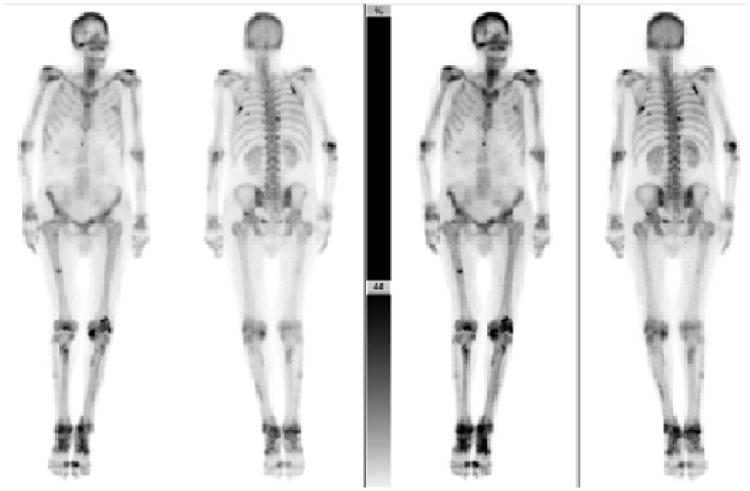
^99m^Tc MDP bone scans from Patient 1 (anterior and posterior) at two different exposure times. Scans showed increased radiotracer in both the axial and appendicular skeletal bones. Darker areas indicate increased radioactive uptake, suggesting periostitis.

Bone scans revealed extensive multifocal bone abnormalities involving both shoulders, sternoclavicular joints, elbows, wrists, hands, knees, ankles and feet. Radiotracer uptake in bilateral tibia and fibula were consistent with periosteal involvement (Figure 1).

The overall clinical picture was consistent with periostitis, secondary to chronic voriconazole use. Voriconazole was promptly stopped, and the patient was started on posaconazole. Over the next several weeks, the patient showed gradual improvement in his joint pain. Approximately one year later, he was seen for orthopedics followup at which time, aside from that associated with longstanding left-knee osteoarthritis, he denied any other joint pain.

### 2.2.Case 2

Patient was a 71 year-old Caucasian male with a history of diabetes and non-ischemic cardiomyopathy who underwent orthotopic heart transplantation. Two months post-transplant, he developed headache, confusion, difficulty with speech, nausea, vomiting and apparent seizure. An MRI of the head showed an irregular ring-enhancing lesion in the left occipital lobe with centrally-restricted diffusion. He underwent exploratory craniectomy. Purulent material was identified within the lesion and removed. Gram staining revealed the presence of septate hyphae consistent with an invasive fungal infection. *Scedosporium* grew in the biopsy sample. The patient was placed on dual therapy consisting of micafungin and voriconazole PO BID. Five days post-initiation, he had a voriconazole trough level of 5.7 mg/L (goal 2 - 6 mg/L). Seven months later, he developed diffuse bone pain. His fluoride level was 26μmol/L (normal range ≤ 4 μmol/L).

**Figure 2:**
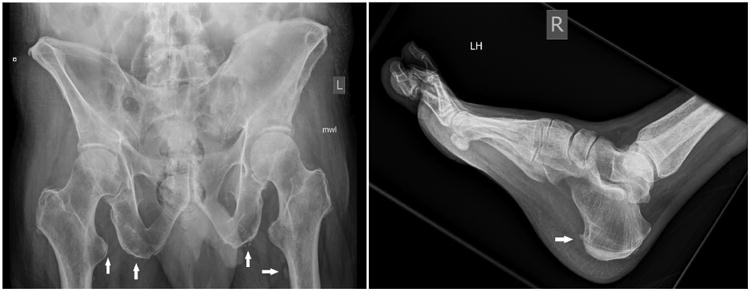
X-rays from Patient 2 (hip left panel; ankle and foot right panel). Films show enthesopathy involving multiple pelvic regions as well as the right heel. White arrows indicate ligamentous ossification characteristic of enthesopathy. Bone eburnation was also noted.

**Figure 3:**
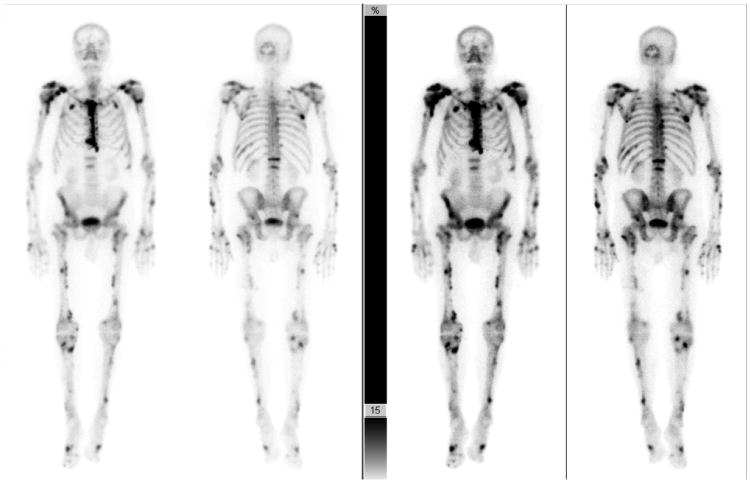
^99m^Tc MDP bone scans from Patient 2 (anterior and posterior) at two different exposure times. As seen in the scans from Patient 1, bone scans from Patient 2 also revealed increased radiotracer accumulations, specifically in the shoulders, ribs, spine and long bones, indicating periostitis.

Musculoskeletal imaging of the abdomen and pelvis revealed mild degenerative changes of bilateral hips, pubic symphysis and sacroiliac joints. There was enthesopathy at the insertion of the right iliopsoas tendon, bilateral gluteus maximus, as well as bilateral hamstring tendons. X-rays of the feet showed a periosteal reaction of both feet involving the second, third, and fourth metatarsals. There was a 7 mm long linear radiopaque dense projection over the right heel, just inferior to the junction of the middle and posterior third of the calcaneus, consistent with enthesopathy (Figure 2), inflammation around the site of insertion of ligaments, tendons, joint capsule, or fascia to bone. Bone scan revealed radiotracer uptake abnormalities throughout the skeletal system including ribs, lumbar spine, scapulae, clavicles, acetabuli, all long bones and scattered bones in hands and feet, consistent with voriconazole-related periostitis (Figure 3).

In this patient, cessation of voriconazole was not a viable option. The isolate was not sensitive to posaconazole. Therefore, he was continued on voriconazole along with a one-year course of micafungin. Within a few months, he was admitted for failure to thrive. During his hospitalization, the palliative care team was engaged in order to optimize his pain management. During evaluation by the team, the patient described an aching pain involving the shoulders, elbows, and chest which he rated as 3 (out of 10) at rest and 8 to 9 (out of 10) with movement. He reported only modest relief with standing acetaminophen and tramadol. Subsequently, he developed bacterial superinfection, gastrointestinal bleeding and cerebrovascular accident, and expired.

## 3. Discussion

Transplant patients on potent immunosuppressants are prone to significant fungal infections. Voriconazole is usually the antifungal treatment of choice for multiple fungal species, including *Aspergilus*, *Scedosporium* and *Fusarium*. However, the present study and the study by Wang and co-workers (2009), demonstrate significant toxicities associated with chronic use of this drug, specifically voriconazole-induced periostitis. Other side effects include mild transient visual disturbance, skin rash and elevated liver enzyme levels [23].

Voriconazole is derived from fluconazole where the triazole ring is replaced with a fluorinated pyrimidine [23]. Drug clearance is primarily dependent on hepatic metabolism. The drug is metabolized in the liver by cytochrome P450 enzymes, particularly CYP2C19. The primary metabolite is voriconazole N-oxide, which has no antifungal property. Approximately 60% of the drug in serum is protein-bound. The bioavailabili-ty of oral voriconazole is >95%. Most of the drug (80%) is excreted in the urine as metabolites.

In our case series, Patient 1 developed joint pain with associated diagnostic changes on imaging, within nine weeks of voriconazole therapy. Patient 1 is the third patient (earlier patients were reported by Becce *et al*., 2012 and Rad, 2015) to develop voriconazole-induced periostitis after a liver transplant [2] [15]. It is likely that hepatic metabolism of voriconazole is affected by the interaction with tacrolimus [24]. Patient 2 was noted to have soft tissue ossification that was discernible in X-rays, in addition to bone changes. The majority of case reports on voriconazole-induced toxicity describe periostitis with nodules or exostoses [9]. Enthesopathy is less commonly observed in voriconazole-toxicity [16]. Since the medication could not be stopped in one of the patients, it is not clear if the soft tissue ossifications would have reversed had the offending agent been discontinued. Since at the time of voriconazole initiation both patients had relatively normal renal function, it is unlikely that impaired renal clearance of the drug was responsible for the development of musculoskeletal complications.

### 3.1.Proposed Disease Mechanisms

The mechanism of voriconazole-induced periostitis and enthesopathy remains unclear. The fluorinated compound theory posits that since voriconazole is a fluorinated compound, over time it releases fluoride, and its subsequent systemic accumulation causes bone toxicity. According to Gerber *et al*. (2012), the World Health Organization has warned that a daily fluoride intake of >6 mg increases the risk for skeletal events. Assuming 96% bioavailability, standard voriconazole dosing can deliver more than ten times that amount [22]. Fluoride is thought to induce new bone formation by stimulating osteoblasts [7] and/or cause inflammation of the periosteum [14]. However, this raises the question as to why all patients with fluoride excess do not develop periostitis. Wermers *et al*. (2011) examined plasma fluoride levels in ten adult post-transplant patients who had received voriconazole for six months, and compared their levels to those who did not receive the drug [4]. All subjects who received voriconazole had elevated fluoride levels (14.32 μmol/L ± 6.41 *vs* 2.54 ± 0.67 μmol/L, treated vs. non-treated respectively; p < 0.001). Discontinuation of voriconazole led to lowering of fluoride levels. These authors hypothesized that impaired renal function may have altered fluoride clearance. However, in our Patient 2 renal function was preserved until his death. Therefore, impaired renal clearance may not be a major player in the development of this disorder. In another study, patients who had received fungal-contaminated methylprednisolone injections were analyzed post-antifungal treatment [22]. Voriconazole treatment was associated with periostitis and pain. These patients reported skeletal pain, and there was a strong association between plasma fluoride concentration and the presence of periostitis. There was no correlation with plasma voriconazole level. Authors concluded that a patient with pain and a plasma fluoride level >8 μmol/L had a greater probability of developing periostitis. Interestingly, patients free of pain and with fluoride levels above 8 μmol/L had a normal bone scan, suggesting that the fluoride test by itself does not have significant clinical predictive value.

Another hypothesis draws upon the similarities between the clinical presentations of voriconazole-induced periostitis and hypertrophic osteoarthropathy (HOA). One factor known to be associated with HOA is the dysfunctional 15-hydroxyprostaglandin dehydrogenase (PGD) gene [3]. Defects in this gene have been reported to cause bone pain, and bone imaging findings are similar to voriconazole-induced periostitis. HOA has been shown to be associated with the upregulation of angiogenic factors. Wang *et al* (2009) suggested that the release of megakaryocytes from the pulmonary vascular bed impacted distal circulation resulting in the release of platelet-derived growth factor (PDGF), which triggers new bone formation [1]. Further investigations into these potential mechanisms are needed.

Finally, hepatic clearance of voriconazole may be important in the development of bone toxicity. Co-treatment with voriconazole and other drugs acting on the cytochrome P450 system, such as calcineurin inhibitors, have raised concern for the rapid progression of voriconazole-induced periostitis [24] [25] [26] [27]. Voriconazole inhibits metabolism of the immunosupressant tacrolimus [24] [25]. Whether impaired voriconazole clearance and/or altered metabolism leads to periosteal changes, needs further study [28]. In 2015, Naito *et al*. reported that inflammation can alter hepatic metabolism of voriconazole [29]. Many transplant patients, including our Patient 2, are usually on steroids for immunosuppression. Steroids can further alter hepatic metabolism of voriconazole. In the clinical cases presented here, both patients had elevated blood alkaline phosphatase levels. We did not fractionate the alkaline phosphatase level to determine the origin of the enzyme, *i.e*. whether hepatic, bone or both. Either way, impaired liver function may play an important role in this condition. As the field of pharmacogenomics advances and personalized medicine becomes the norm, individual drug disposition and rare toxic effects in specific subpopulations of patients will need to be carefully assessed [30].

### 3.2.Summary of Management Recommendations

The diagnosis of voriconazole-induced periostitis and enthesopathy should be strongly considered in transplant patients receiving the drug as the antifungal treatment. The CDC recommends therapeutic blood voriconazole trough levels of 2 - 5 μg/mL [11]. Based upon the literature, patients on voriconazole treatment for greater than two months, particularly if receiving daily doses exceeding 500 mg a day, [11] should be assessed for musculoskeletal pain. Along with pain evaluation, a thorough investigation should include measurement of alkaline phosphatase, voriconazole trough, and fluoride levels, and a bone scan. If fluoride levels exceed >8 μmol/L and voriconazole continuation is deemed a clinical necessity, a fifty percent dose reduction is advised in order to lower the fluoride level to a maximum of 8 μmol/L. Even if the decision is made to discontinue voriconazole due to periostitis, with or without enthesopathy or exostoses, monitoring of fluoride and alkaline phosphatase levels is advised for up to six months, particularly when other hepatically-cleared medications are administered. Early recognition of voriconazole-induced musculoskeletal toxicity is critical for the early withdrawal of the offending agent.

## 4.Conclusion

Here we present case reports of two solid organ transplant patients who developed voriconazole-induced periostitis and enthesopathy. Symptoms included severe bone pain, elevation in fluoride and alkaline phosphatase levels, and abnormal musculoskeletal imaging, without any confounding rheumatologic diagnosis. Voriconazole-induced periostitis can result from fluoride toxicity, abnormalities in the pulmonary vascular bed leading to the production of downstream inflammatory mediators, or abnormal pharmacokinetics of hepatic drug metabolism. These mechanisms need to be examined in the future.

## Figures and Tables

**Table 1:** Clinical and laboratory characteristics of solid organ transplant patients with voriconazole-induced periostitis and enthesopathy.

	Patient 1	Patient 2
**Solid Organ Transplant (Indication)**	Liver (HCV)	Heart (Non-ischemic)
Age at transplant, y	56	70
Immunosuppression regimen	Tacrolimus, MMF	Tacrolimus, MMF, prednisone
**Sex**	Male	Male
Body mass index, kg/m2	27.9	30.2
Voriconazole exposure time leading to periostitis	61d (8.7 wks)	257d (36.7 wks)
Estimated total voriconazole dose until onset, g	48.4	205
Mean tacrolimus trough level, ng/mL	5.3	8.9
Median tacrolimus trough level, ng/mL	4.8	8.7
**Laboratory values at voriconazole initiation**		
Glomerular filtration rate, mL/min	58	>60
Alanine transferase, U/L€	13	14
Alkaline phosphatase, U/L¥	107	75
Parathyroid hormone, pg/mlΩ	13	N/A
**Laboratory values during voriconazole treatment**		
Glomerular filtration rate, mL/min	∼20*	>60
Alanine transferase, U/L€	9	48
Alkaline phosphatase, U/L¥	132	1090
Parathyroid hormone, pg/mlΩ	N/A	72
Voriconazole level, trough, max/normalization, mg/L®	N/A	7.7/4.3
Fluoride level, peak/normalization∞, umol/L	24.6/5.8	28.3/UD

€ = normal range 45 - 115;

¥ = normal range 10 - 55;

Ω = normal range = 10 - 65;

® = goal range 2 - 6;

∞ = normal range = 0 - 4;

Normalization refers to remote period after drug removal or dose reduction.

* indicates transient dialysis dependence;

UD = undetectable.
